# A new hazard assessment workflow to assess soil contamination from large and artisanal scale gold mining

**DOI:** 10.1007/s10653-023-01552-5

**Published:** 2023-04-18

**Authors:** Matar Thiombane, Benedetto De Vivo, Birane Niane, Michael J. Watts, Andrew L. Marriott, Marcello Di Bonito

**Affiliations:** 1 Haemers Technologies Group, Chaussée de Vilvorde, 104, 1120 Brussels, Belgium; 2 Pegaso University, Piazza Trieste E Trento 48, 80132 Naples, Italy; 3 Virginia Tech, Blacksburg, VA 24061 USA; 4 Départment Génie Géologique, Mines Et Eau, U.F.R. Sciences de L’Ingénieur, Université IBA DER THIAM de Thiès, BP 967, Thiès, Senegal; 5 Inorganic Geochemistry, Centre for Environmental Geochemistry, British Geological Survey, Keyworth, NG12 5GG UK; 6 School of Animal, Rural and Environmental Sciences, Nottingham Trent University, Brackenhurst Campus, Southwell, NG25 0QF UK; 7 Department of Agricultural and Food Sciences, AlmaMater Studiorum-University of Bologna, Via Fanin, 40, 40127 Bologna, Italy

**Keywords:** Potentially harmful elements (PHE), Geochemical signatures, Contamination level, Risk assessment, human health

## Abstract

Gold mining activities are undertaken both at large and artisanal scale, often resulting in serious ‘collateral’ environmental issues, including environmental pollution and hazard to human and ecosystem health. Furthermore, some of these activities are poorly regulated, which can produce long-lasting damage to the environment and local livelihoods. The aim of this study was to identify a new workflow model to discriminate anthropogenic versus geogenic enrichment in soils of gold mining regions. The Kedougou region (Senegal, West Africa) was used as a case study. Ninety-four soil samples (76 topsoils and 18 bottom soils) were collected over an area of 6,742 km^2^ and analysed for 53 chemical elements. Robust spatial mapping, compositional and geostatistical models were employed to evaluate sources and elemental footprint associated with geology and mining activities. Multivariate approaches highlighted anomalies in arsenic (As) and mercury (Hg) distribution in several areas. However, further interpretation with enrichment factor (EFs) and index of geoaccumulation (IGeo) emphasised high contamination levels in areas approximately coinciding with the ones where artisanal and small scale mining (ASGM) activities occur, and robust compositional contamination index (RCCI) isolated potentially harmful elements (PHE) contamination levels in very specific areas of the Kedougou mining region. The study underlined the importance of complementary approaches to identify anomalies and, more significantly, contamination by hazardous material. In particular, the analyses helped to identify discrete areas that would require to be surveyed in more detail to allow a comprehensive and thorough risk assessment, to investigate potential impacts to both human and ecosystem health.

## Introduction

Increasing world population and technological advancement requires mineral exploration of valuable ore resources to be at the forefront of economic investments, both for technological advance as well as manufacturing. In the last few decades, exploration geochemistry has enabled the exploitation of ore deposits worldwide thanks to advanced spatial and geostatistical methods that accurately identify geochemical anomalies (Cheng et al., 1994, 2000; Lima et al., 2003; Zuo et al., 2015). However, the same methodologies can provide vital information on contamination and potential risk to human health, representing a rare case of a methodology that can be used as tool for both development and sustainability (Albanese et al., 2010; Minolfi et al., 2017, Nazarpour et al., 2018). Exploration and mining of gold (Au) is undertaken at large or artisanal scale in many regions of the world. Gold mining activities can result in environmental issues, including environmental pollution and hazard to human and ecosystem health, which if not regulated can induce long-lasting damage to the environment and local livelihoods. In particular, soil pollution induced by large scale gold mining (LSGM) can be a threat to wildlife and human health. Frequently, valuable Au ores occur in rocks containing concentrations of sulphides of various elements (e.g., As, lead—Pb, copper—Cu, and Hg), whose exploitation results in the generation of oxidizing conditions and hence environmental risk, due to sulphuric acid release. Washing these toxic by-products away results in semi-solid slurry called “tailings”, which contaminate the soil and other environmental matrices (Appleton et al., 2001; Kaninga et al., 2019; Naicker et al., 2003; Ogola et al., 2002; Rivera-Parra et al., 2021; Sako & Nimi, 2018). Furthermore, even at relatively low concentrations, the presence of such trace elements in food can have significant harmful effects on human health, amplified by the potential for bioaccumulation in the food chain (Derakhshan et al., 2018; Duruibe et al., 2007; Marriott et al., 2019; Sadeghi et al., 2022; Shams et al., 2022; Watts et al., 2013). Artisanal and small-scale Au mining (ASGM) continues to grow in the developing world, creating thriving local rural economies but also causing significant environmental contamination and health issues. The ASGM is particularly problematic as it involves the use of Hg in the Au extraction process (Van Straaten, 2000; Campbell et al., 2003; Veiga et al., 2006). The extensive use of Hg to amalgamate Au by artisanal miners leads to direct emissions of Hg into the atmosphere as well as its release to soil and water in various forms (Cordy et al., 2011; Grandjean et al., 1999; Malm, 1998; Veiga et al., 2006). As reported by the World Health Organisation (WHO) in their guidance for identifying populations at risk from Hg exposure (WHO, 2003) and with the Minamata convention (UNEP, 2013), some of these activities are particularly common in developing countries, where regulations, environmental controls and monitoring tend to be limited, producing risk 'hot spots' from exposure. For this reason, identifying potential risks in both large- and small-scale Au mining activities is of crucial importance both to protect the ecosystem services and the health and well-being of the communities affected.

Spatial and geostatistical computations have been used to distinguish the source of Potentially Harmful Elements (PHE) related to mining activities. For example, various fractal/multifractal methods (e.g., Concentration-Area) have been given particular attention in mineral exploration, environmental health-risk analysis and regional topsoil studies to display a spatial distribution of elements (Agterberg, 2001; Cheng, 1999; Cheng et al., 1994; Lima et al., 2003; Zuo & Wang, 2015; Zuo et al., 2015). Geostatistical methods (e.g., regression analysis or factor analysis) have enabled the identification of relationships between the elements and their possible source patterns (Albanese et al., 2015; Petrik et al., 2018a, 2018b; Reimann et al., 2002; Thiombane et al., 2019). Various contamination indices have been developed and used to characterise pollution or contamination of different environments; some examples are the Enrichment factors (EF, Chester & Stoner, 1973) and the Geoaccumulation Index (Müller, 1969). In many cases, these indices are relatively simple to implement as they are using reference values from background/baseline values (e.g., Enrichment factor) which are readily available from the literature (Atapour, 2015; Zuzolo et al., 2017; Liu et al., 2018; e.g., Seifi et al., 2019; Xu, Sun and Wang, 2019; Chen et al., 2020). Nevertheless, issues have been raised with regards to their robustness in situations where changes in concentration units occur, leading to alterations in the results of the analysis (i.e., not scale-invariant, see Aitchison & Egozcue, 2005; Pawlowsky-Glahn et al., 2015 for more details). For this reason, we have also used a Robust Compositional Contamination Index (RCCI) that follows the compositional structure of geochemical data (Jarauta-Bragulat et al., 2015; Petrik et al., 2018a).

This study aims to reveal the source patterns of PTEs by using a combination of multivariate analyses and GIS approaches, as well as assessing the contamination level of 15 PHEs in the soils of the Kedougou region (Senegal). The results from this investigation will provide a blueprint for future studies and greatly improve our understanding of the environmental processes linked to these anthropogenic activities. Furthermore, since it is based on the first geochemical topsoil survey of the region, this study will be of value in establishing fundamental guidelines, e.g., background/baseline values for these toxic elements and push towards an institutional response for a more adequate and comprehensive regulations in Senegal.

## Materials and methods

### Features of the survey area

**Fig. 1:**
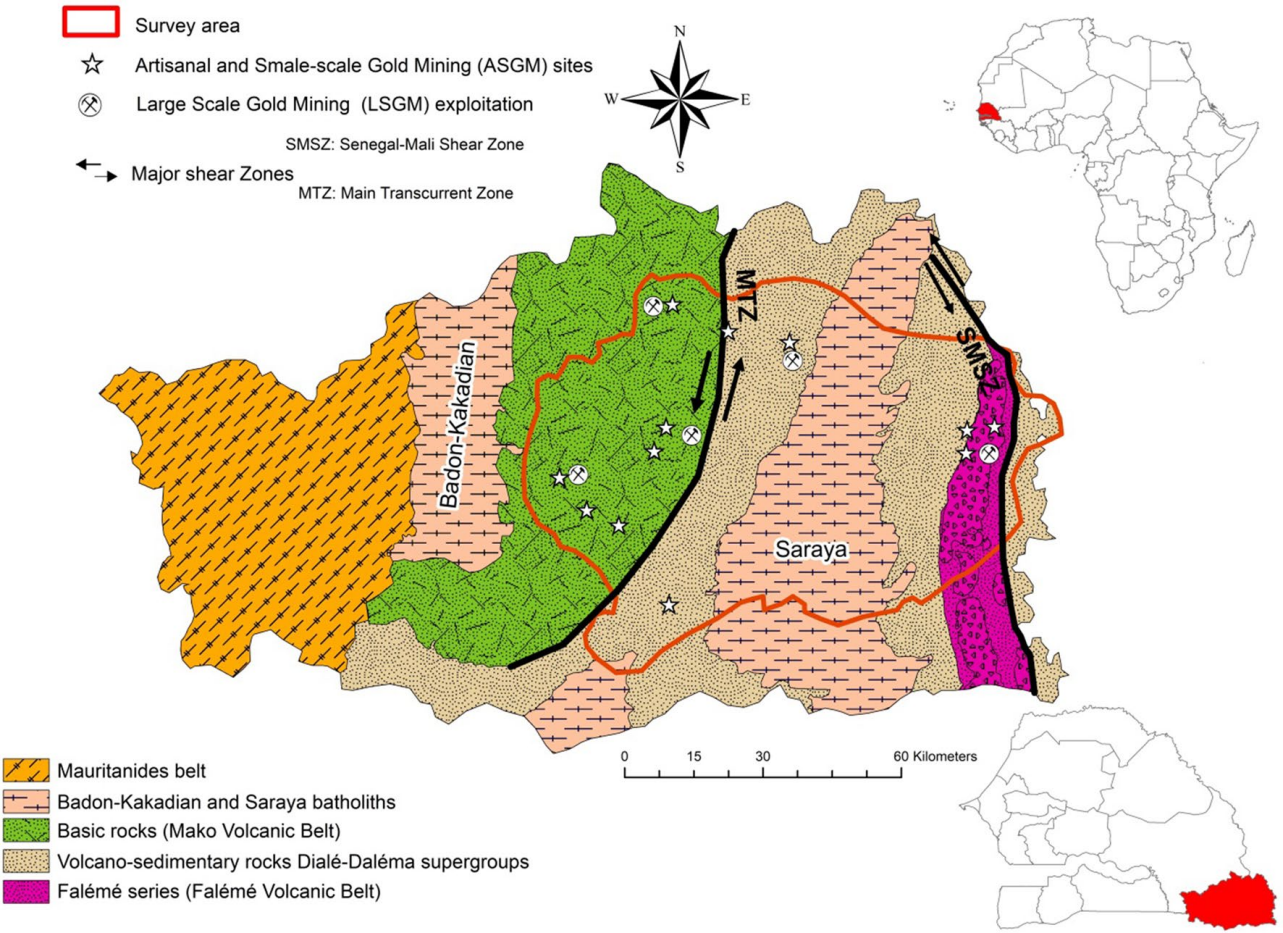
Main geology and lithology of the Kedougou Kénieba inlier; the map represents the part settling in the Kedougou region (modified after Lawrence (2010)

The Kedougou Region is situated in the south-eastern part of Senegal and occupies a territory of about 16,800 km^2^. The region borders Mali and the Guinee Bissau on the eastern and southern sides, respectively. The main geological features of the region are constituted of a Precambrian basement formed by the so-called Kedougou-Kénieba inlier (Bassot, 1987). This consists of volcanic, volcano-sedimentary and sedimentary formations, which are distributed into two Supergroups: the Mako Supergroups or “Mako Volcanic Belt” to the West and the Dialé-Daléma Supergroups in the East (Fig. 1). The geological formations from these two Supergroups serve as hosts to several generations of granitoids in coalescent solid masses distributed into two batholiths, Badon-Kakadian and Saraya, intruding the Mako and Dialé-Daléma Supergroups, respectively.

The “Falémé Volcanic Belt” outcrops to the east of the Daléma basin are dominated by plutonic rocks, the Balangouma and the Boboti complexes in the centre and south of the belt, respectively (Hirdes & Davis, 2002; Lambert-Smith et al., 2016; Lawrence et al., 2013).

**Fig. 2:**
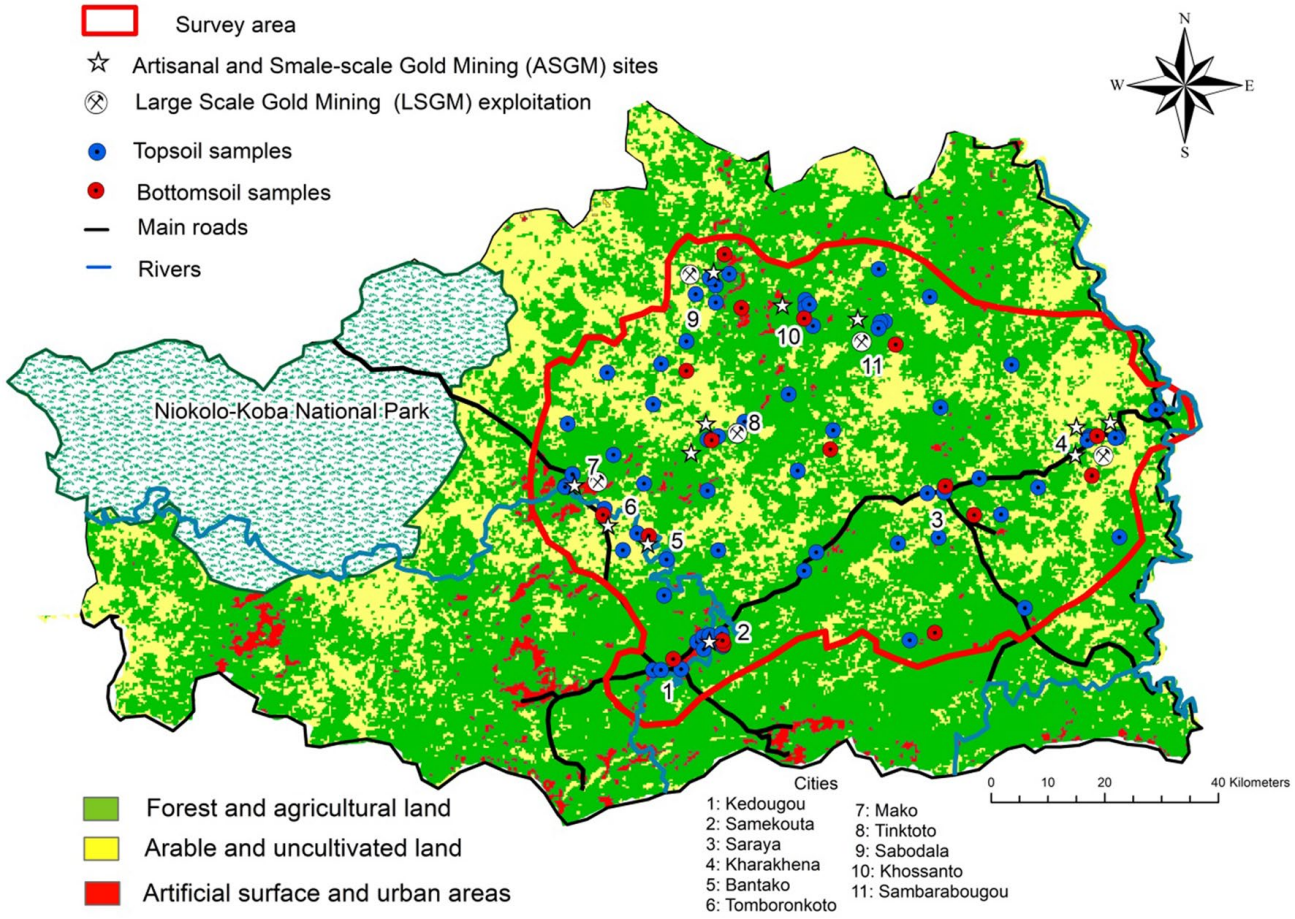
Land use, hydrographic network and soil samples locations in the study area

These ancient lands, commonly called "Birimian formations", constitute a metallogenic province of great importance, bearing different ore deposits and Au-bearing reserves. The main Au deposits in the Kedougou region are related to the major tectonic zones, namely: the Main Transcurrent Zone (MTZ) and the Senegalo–Malian Shear Zone (SMSZ) (Fig. 1), which are major regional controls of Au mineralisation (Bassot, 1987; Sylla & Ngom, 1997). There are two types of Au occurrences in the study area: (1) alluvial Au, which occurs mainly in the hydrographic network and (2) Au-bearing quartz veins hosted by shear zones. The Kedougou region is an emerging significant Au field where the Teranga Gold Operation (TGO) is currently the largest active mining company exploiting the shear zone hosting Au-bearing quartz veins. In addition, several other Au mining companies are in prospection/exploitation steps such Randgold-Sénégal company, Toro Gold, Bassari resources and Oromine companies. Moreover, many ASGM activities are scattered throughout the surveyed area (Fig. 2).

The relief of the region is the most rugged of the country with a peak of 581 m in the Southern part. Kedougou has one of the highest rainfalls in the country with at least 1300 mm / year (ANDS, 2015). The wet season lasts approximately 6 months, from May to October. It should be noted that tropical ferruginous soils and poorly evolved soil erosion predominate in the region. Such a situation results in this area being very important for biodiversity both from a faunal and a floristic point of view. The region is one of the last strongholds of Senegal's fauna composed of the most prestigious antelope bands of the world as well as buffaloes, lions, leopards, and crocodiles, occurring predominantly in the largest natural park of the country, the Niokolo-Koba National Park (Fig. 2). In general, almost all the species of the Guinean flora are present in Senegal but also a good part of the fauna of West Africa. This rich biodiversity is partly the result of favourable climatic characteristics as well as geomorphological features (ANDS, 2015).

The hydrographic network is dense and depends very heavily on rainfall. The area is drained by the Falémé and the Gambia rivers and their large number of tributaries. The Gambia and Falémé, which constitute the two major rivers, play an important role in local development (agriculture, water supply for men and livestock) (Fig. 2).

With a population of 156,352 inhabitants in 2017, the region remains sparsely populated, representing only 1.2% of the population of Senegal with a density of 9.1 inhabitants per km^2^ (ANDS, 2015).

The Kedougou region has significant mining potential, making it highly attractive for investors. Mining activities occupy a high proportion of the population of the region and large groups are involved in Au panning and ASGM activities. During the last few years, mining activities have boosted the economic conditions of this region which previously was only embryonic. Gold mineralisation is the main ore deposit that is being exploited industrially and through ASGM and LSGM activities. The National Agency of statistics and demography of Senegal (ANDS, 2015) has outlined 29 ASGM locations in the Kedougou region and most of them use Au-Hg-panning methods on the banks of Gambia and Falémé rivers and their tributaries (Fig. 2). Consequently, river-Hg-contamination is likely to constitute a real concern for population living in these areas consuming fish from the rivers (Counter & Buchanan, 2004; Ishikawa & Ikegaki, 1980). The Hg from geogenic and anthropogenic (e.g., ASGM for Au amalgamation) activities can accumulate in sediments following a combination of hydrological and geomorphological mechanisms such as surface runoff and inflow, wet and dry deposition. Furthermore, under anaerobic conditions, bacteria can transform Hg through methylation and methylmercury (CH_3_Hg) could bio-accumulate in the biota and enter the food chain. Human populations living in the proximity of the artisanal Au mining areas would therefore be more susceptible to Hg exposure, which is considered neurotoxic and teratogenic for humans (WHO, 2003; UNEP, 2013). Furthermore, associated problems of over-exposure of Hg with other metals such as Zn, Cu, and aluminium (Al) are gastrointestinal (GI) disorders, diarrhoea, stomatitis, tremor, hemoglobinuria, ataxia, paralysis, skin lesions, vomiting and convulsions (Marriott et al., 2019; Singh et al., 2018).

### Soil sampling and analytical procedures

The fieldwork was organised from early March to the end of April 2018 to collect the most representative soil samples in the study area (Fig. 2). Based on the specific land-use, geological features and the emplacement of large-and small-scale gold mining activities, in 6,742 km^2^, a specific sampling design was produced where a total of 76 topsoil samples at a depth between 5 and 20 cm (after vegetation cover was removed) and 18 bottom soils (1–3 m) were collected. A higher soil sampling density (1 sample per 15.5 km^2^) was planned in surrounding urban, LSGM and ASGM areas, and a lower one (1 sample per 79.5 km^2^) in unused or arable land. This specific sampling design aimed to reveal better element patterns and their possible pollution sources mostly in industrial and/or urbanized areas.

The soil sampling was carried out following the Geochemical Mapping of Agricultural and Grazing Land Soils (GEMAS) procedure (Reimann et al., 2014). Topsoil samples (from 5 to 20 cm) were created by homogenizing 5 subsamples at the corners and the centre of a 100 m^2^ square, resulting in approximately 1.25 kg in total. Bottom soil samples (1–3 m) were collected mostly nearby unused and remotes sites where there were no human activities, in order to allow an assessment of the background/baseline concentrations of elements in the soils of the study area (i.e., using them as ‘control sites’ and look at their relations to the parent material).

The following soil properties were measured and recorded by geospatial positioning systems (GP at each site: pH, total water content, conductivity, total organic content, as well as visual observations on colour, landuse, and vegetation cover). The 94 samples were prepared following the procedure described in Thiombane et al. (2018): after drying under infra-red lamps at *T* < 35 °C, samples were pulverized in a ceramic mortar and sieved to retain the < 2 mm fraction, and stored (30 g) in individually labelled plastic bags for chemical analysis.

Chemical analyses for 53 elements followed procedures as described in Petrik et al. (2018b): and were carried out by an accredited laboratory Acme Analytical Laboratories Ltd. (now Bureau Veritas, Vancouver, Canada). The samples were analysed by both inductively coupled plasma atomic emission (ICP-AES) and inductively coupled plasma mass spectrometry (ICP-MS—Perkin Elmer Elan 6000/9000) for “pseudototal” concentration to estimate mobile PHEs (Adamo & Zampella, 2008; Vercoutere et al., 1995) after digestion by the ultratrace aqua regia extraction method (Bureau Veritas, 2017). Whilst aqua regia does not dissolve primary silicates, trace elements associated with most other major soil components are released.

A subsample (15 g) was digested in 90 mL aqua regia and leached for 1 h at 95 °C in a water bath. After cooling, the solution was diluted to 300 mL with 5% hydrochloric acid (HCl), for a sample weight to solution volume ratio of 1 g per 20 mL. The accuracy and precision of the data were assessed against known analytical standards. Precision (median value of the Relative Percentage Difference, RPD) of the analysis was calculated using three in-house replicates, and two blind duplicates submitted by the authors. Accuracy is calculated on Bureau Veritas’s in-house reference materials (STD DS9, STD DS10, STD DS11, and STD OXC109). Reference materials were calibrated to an aqua regia digestion/ICP-MS determination against published values for a concentrated HCl and nitric acid (HNO_3_) digestion of the Canadian Certified Reference Materials Project (CCRMP) TILL-4 and LKSD-2.

### Data analysis and workflow model

**Fig. 3:**
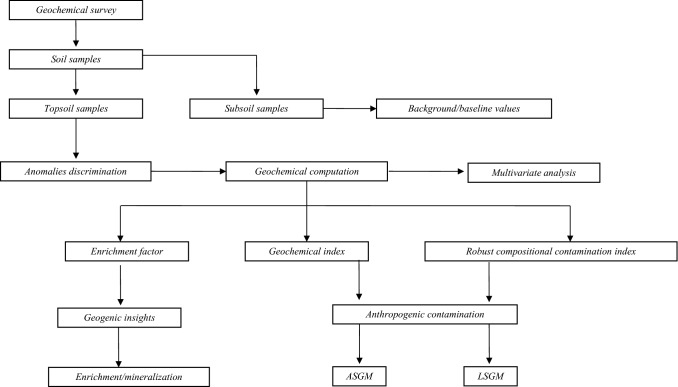
The flowchart of geochemical survey to discriminate the sources and contamination level of the 15 PHEs in the study area

A new workflow was designed to guide the geochemical survey, in order to better understand the main geochemical processes that control elements distribution and to discriminate the sources and contamination level of 15 PHEs (Cu, Pb, Zinc—Zn, Nickel—Ni, Cobalt—Co, As, Cadmium—Cd, Antimony—Sb, Vanadium—V, Chromium—Cr, Thallium—Tl, Hg, Selenium—Se, Tin—Sn, and Beryllium—Be) in the study area (Fig. 3). These 15 elements, which are listed as PHEs by the Italian legislation (D. Lgs. 152/2006), represent one of the foremost environmental issues, and their high concentrations put humans at risk.

#### Mapping and spatial statistics

A frequency-based method (scatterplot and factor analysis) and frequency space-method (spatial display) were used to visualize element distribution in the survey area: these methods have shown to be powerful tools to assess the features of elements and highlight possible geogenic or anthropogenic contributions (Reimann et al., 2005; Albanese et al., 2007; Petrik et al., 2018b).

In order to consider local variability and singular property as well as spatial association, the GeoDAS™ software was used to produce interpolated geochemical maps. The fractal/multifractal inverse distance weighted (MIDW) algorithm was applied for these computations (Cheng et al., 1994; Lima et al., 2003). Previous work established that MIDW maintains high frequency information as well as retaining local variability related to both locational connotations and local singularity (Cheng, 1999; Cheng et al., 1994; Lima et al., 2003; Petrik et al., 2018b). Singularity describes the scaling dependency from a multifractal point of view by using an index, which measures the behaviour of spatial variables changes with measuring scale changes (Cheng, 1999; Cheng et al., 1994). Locational connotation (or spatial association) describes the statistical dependency of values at separate locations, and its indices (e.g. covariance, autocorrelation and semivariogram) characterize the local structure and variability of surfaces (Cheng et al., 1994).

Concentration intervals are classified based on the concentration–area (C–A) fractal plot (Cheng, 1999; Cheng et al., 1994, 2000) and ArcGIS™ were used for the spatial presentation of the results, to allow the capture of the different patterns controlling element behaviour.

Frequency-based method (graphical displays) were implemented by using the open source statistical software *R* version 4.0 (Templ et al., 2011; Van Den Boogaart et al., 2011) and data log-transformations by means of another open source software, CoDaPack (Comas-Cufí & Thió-Henestrosa, 2011).

Based on the work by Thiombane et al. (2019), the number of elements for statistical computations was reduced to a smaller number of 28 variables based on two criteria: 1) the removal of elements with > 50% of values below the detection limit (LOD) and 2) choosing elements with a communality of extraction > 0.5 (50%) and/or common variances < 0.5 (e.g., Reimann et al., 2002).

#### Multivariate statistics

Factor analysis (FA) was used to elucidate the correlation structure of the variables by aggregating them within fewer factors (Reimann et al., 2002). Factor analysis has been shown to be successful in identifying element sources and relate them to their main potential origins (Reimann et al., 2002; Albanese et al., 2007; Petrik et al., 2018b; Reyes et al., 2020). An isometric logratio transformation (ilr) was applied on raw-data prior to multivariate analysis (Filzmoser et al., 2009) to minimize or eliminate the presence of outliers and spurious correlation (Pawlowsky-Glahn and Buccianti, 2011). In order to facilitate the interpretation of results, varimax (orthogonal) rotation was implemented; this was shown to curtail the number of variables with high loadings on each factor, streamlining and improving the interpretation of the transformed and simplified data matrix (Reimann et al., 2002; Petrik, 2018b). Factor analysis in R-mode was used to obtain aggregate factors to be interpreted based on their potential origin (i.e. geogenic, anthropogenic or mixed).

#### Multivariate regression and discrimination analysis

Based on the FA and element associations, a discrimination regression was performed using four variables: As, Hg, Sb, and Au. Discrimination analysis is widely used in geostatistical computations to highlight the group of elemental behaviour and further distinguish between geogenic enrichment and anthropogenic contamination (Reimann et al., 2005; Petrik et al., 2018b). Arsenic and Hg ratio was used as independent variable as these two elements may better distinguish and discriminate ASGM and LSGM activities in the study area. Based on the high correlation between As and Sb, and between Sb and Au, the Sb/Au ratio was computed as a dependent variable.

The regression plot was divided into several parts based on the classification of ratios by means of Tukey's box-and-whisker plots. The area below the lower quartiles (*Q*_1_) was identified as ASGM, where Hg and Au elements prevail, whilst the area above the upper quartiles (*Q*_3_) was identified as LSGM given the high predominance of As and Sb variables. In this way, it was possible to discriminate elemental source patterns associated with an ASGM or/and LSGM activities.

### Geochemical computations

Several geostatistical computations are available in the literature to distinguish mineralisation from contamination (Cheng, 1999; Cheng et al., 1994; Lima et al., 2003; Reimann & De Caritat, 2005). The following sections describe the ones used in this study to discriminate between underlying geological features and/or anthropogenic activities.

#### Enrichment factor

The Enrichment Factor (EF) approach was first introduced to recognise economically viable mineralisation and has been used to identify the origin of elements in the atmosphere and in precipitation including seawater (Goldberg, 1972; Chester & Stoner, 1973; Peirson et al., 1974; Duce et al., 1975; Rahn, 1976; Buat-Ménard & Chesselet, 1979). In this study, it was applied to determine soil contamination on a long-term scale (Abrahim & Parker, 2008; Hakanson, 1980; Saeedi et al., 2012; Sutherland et al., 2000; Thiombane et al., 2019; Wu et al., 2011). Enrichment Factor was calculated using the same equation, introduced by Chester and Stoner (1973—Eq. (1) below) as follows:

1 $${\text{EF}} = \frac{{\left( {\frac{Cx}{{Cref}}} \right){\text{sample}}}}{{ \left( {\frac{Cx}{{Cref}}} \right){\text{background}}}}$$

with *C*_x_ representing the concentration of the element to calculate and *C*_ref_ the concentration of a reference element. The background concentrations of the considered PHEs were determined in 18 bottom soil samples collected in areas characterised by an absence of industrial activities. Moreover, their respective Geometric Means (GeoM) were considered as background concentrations over arithmetic mean and median because GeoM is a robust index that minimises/eliminates the influence of “outlier observations” throughout the data analysis. Here, the reference element is an element that is particularly stable in soil, which is validated by its vertical immobility and/or chemical stability (non-degradability) (Reimann et al., 2008). Aluminium, Sc, Zr and Ti, naturally occurring in soils, are the main elements considered to be stable. The EF is represented in five numerical ranges, each corresponding to an increasing level of pollution (Sutherland, 2000). In this study, the choice of the most stable element in EF calculations was based the coefficient of variation (CV) as presented in Thiombane et al. (2019), which can be calculated using the equation:

2 $${\text{CV}} = \frac{{{\text{MAD}}}}{{{\text{MD}}}} \times 100\%$$

where CV is the variability of distribution in percentage (%), MAD is the median absolute deviation, i.e. the 50th percentile of the deviations of all concentrations from the median value of concentration, and MD is the median concentration. This robust, nonparametric estimate is not affected by the presence of outliers and allows a more extensive interpretation of the variability of distribution of reference elements (Reimann & de Caritat, 2005). The smaller the CV value, the more stable the element will be.

#### Index of geo-accumulation

The geo-accumulation index (IGeo) was originally introduced by Müller (1969) to quantify accumulation in sediments. In recent years it has been used in different fields (Kowalska et al., 2016; Varol, 2011) to assess PHEs pollution level on the basis of the ratio between the current content of the element in topsoil and the background content of the element. The Geo-accumulation index is expressed as follows:

3 $$I{\text{Geo}} = \log 2\left( {\frac{Cx}{{1.5Cb}}} \right)$$

where *C*_x_ is the concentration of the element under consideration and *C*_b_ is geochemical background value of an element. Factor 1.5 is the background matrix correlation factor due to the lithological effects. The IGeo values are usually classified in seven numerical ranges, each representing a pollution level (Müller, 1969).

#### Robust compositional soil contamination index (RCCI)

The above-described indices, as well as others available in the literature, (e.g., Chester & Stoner, 1973; Müller, 1969; Hakanson, 1980) use background/baseline values for reference and are relatively intuitive. Nevertheless, in recent years these have been critically reviewed as they appear to over-simplify very complex processes. As reported by Petrik et al. (2018a), reasoned that their values can strongly depend on the parent rock materials and selected reference media as well as reference elements, which can obviously vary significantly in various contexts (Reimann and de Caritat 2000, 2005). Furthermore, other authors have highlighted that they are not scale-invariant, and that the results of the analysis may change depending on the concentrations units used (Aitchison & Egozcue, 2005; Pawlowsky-Glahn et al., 2015). Arguably, these simplifications can be justified by the PHEs long biological half-lives and their tendency to bioaccumulate in soils by adsorption to clay minerals and organic matter (Kabata-Pendias, 2011). On the other hand, PHE bioavailability can be dictated by various physicochemical factors (e.g., pH, Eh) as well as physiological variations (Barkouch et al., 2007; Kabata-Pendias, 2011; Skordas & Kelepertsis, 2005; Yang et al., 2004; Zhao et al., 2013, 2014). For these reasons, in this study the standard indices have been coupled with a more comprehensive calculation, the robust compositional soil contamination Index (RCCI) which tries to identify the pollution level taking into account the compositional character of the data (Petrik et al., 2018a; Reyes et al., 2019). The RCCI can be expressed following three different steps. The first step looks at characterizing each individual sampling site as follows:

4 $$X_{i} = C_{i} /B_{n}$$

with *X*_i_ the ratio between the concentration of the metal (*C*_i_) and the geochemical background/baseline (*B*_n_) calculated for each element at each sampling site *i*. In this study, the baseline was calculated considering the geometric mean (GeoM) of each PHE from bottom soils sampled in areas not affected by the mining operations (control sites). GeoM reflects the central tendency of the dataset and it is not affected by the presence of outliers. At the end of this step, a series of Xi values is obtained for each element and for each site, reflecting the individual elemental ratios at that specific location. In the second step, each site is treated in a ‘compositional’ way, producing a weight for that site with respect to the entire dataset, including all element considered as follows:

5 $$Z_{i} = {\text{ Geo}}M\left( {X_{i} } \right)$$

where the weight *Z*_i_ is obtained by calculating GeoM for each sampling location constituted of all the elemental *X*i previously calculated. This will therefore provide an individual weight *Z*i for each sampling site, but in a compositional manner. The third step will look at the various *Z*i and consider how they vary from the maximum *Z*, providing a ‘compositional enrichment’ interpretation. This will equate to:

6 $${\text{RCCI}} = \, \left( {Z_{i} /Z_{\max } } \right)x100\%$$

where RCCI is the ratio between *Z*_i_ and *Z*_max_, the geometric mean of the sampling point *i* and the maximum geometric mean, respectively. The results are expressed in percentages from 0 to 100% with increasing degree of contamination reflected by RCCI values nearer to 100%. For a better understanding of the origins of this index, as well as the assumptions considered for its application on soils, please refer to the robust index introduced by Jarauta-Bragulat et al. (2015) to report Air Quality Index (AQI) of the city of Madrid (Spain) and the subsequent work by Petrik et al (2018b) to adapt it to soils.

## Results and discussion

### Variation of element concentrations

Table 1 shows the variation and descriptive statistics of investigated elements. The distribution of the 28 considered elements are mainly right-skewed, which indicates that the mean value is typically greater than the median.

**Table 1:** Descriptive statistic of 76 topsoils samples from the Kedougou region (South-eastern of Senegal; CV and Std. Deviation are the coefficient of variation and standard deviation, respectively

Elements (mg kg^−1^)	Minimum	Maximum	Geometric mean	Median	Std. deviation	Skewness	Kurtosis	CV (%)
Ag	0.003	1.09	0.06	0.03	0.14	5.85	38.5	44.2
Al	1900	27,300	11,805	11,300	6457.8	0.2	− 0.8	30.1
As	0.2	121.8	15.43	6.3	21.1	2.89	10.47	73
Au	0.0001	7.72	0.37	0.01	1.21	4.56	21.7	1629
Be	0.06	1.3	0.62	0.6	0.35	0.2	− 1.04	50
Ca	65	14,500	2640	1400	3425	2.2	4.17	786
Cd	0.01	0.28	0.03	0.01	0.05	3.06	10.29	42.9
Co	0.5	61.8	12.77	7.6	13.23	1.69	3.07	132
Cr	13.2	770.6	151.17	114.4	149.34	2.07	4.59	37
Cu	2.69	92.26	27.89	23.89	20.3	0.89	0.3	6.2
Fe	3700	265,200	63,801	48,400	53,578	1.28	2.09	7
Hg	0.003	2.4	0.14	0.03	0.36	4.33	20.52	75
K	200	3000	740	600	526	1.67	3.57	400
Mg	65	3600	1041	800	882	1.33	1.19	350
Mn	26	2122	433	294	414	1.97	4.57	59
Mo	0.15	21.3	1.93	1.08	3.08	4.22	20.96	40
Na	6.5	240	42.51	30	41.68	2.23	6.12	67
Ni	1.6	96.4	22.73	14.8	22.38	1.41	1.23	106
P	40	1550	365	340	289	1.41	2.8	47
Pb	1.77	25.37	9.28	8.28	5.56	0.79	0.27	44
Sb	0.02	5.6	0.69	0.41	0.85	3.08	13.72	66
Se	0.06	0.9	0.21	0.06	0.22	1.54	1.24	823
Sn	0.3	2.8	0.7	0.6	0.44	2.61	8.7	33
Th	0.8	8.5	3.65	3.1	1.79	0.73	− 0.23	55
Ti	60	920	297.9	260	183.9	1.08	1.52	4
Tl	0.01	0.19	0.05	0.04	0.03	1.75	3.23	50
U	0.2	3.8	1.05	0.9	0.7	1.69	3.76	22
V	12	645	131.49	110	110.8	1.63	4.49	25
Zn	2.3	374.2	35	20.8	55.01	4.42	22.05	19

Spatial distribution of the elements was interpreted by producing interpolated maps. In particular, As and Hg concentration variations were investigated in detail in the study area.

**Fig. 4:**
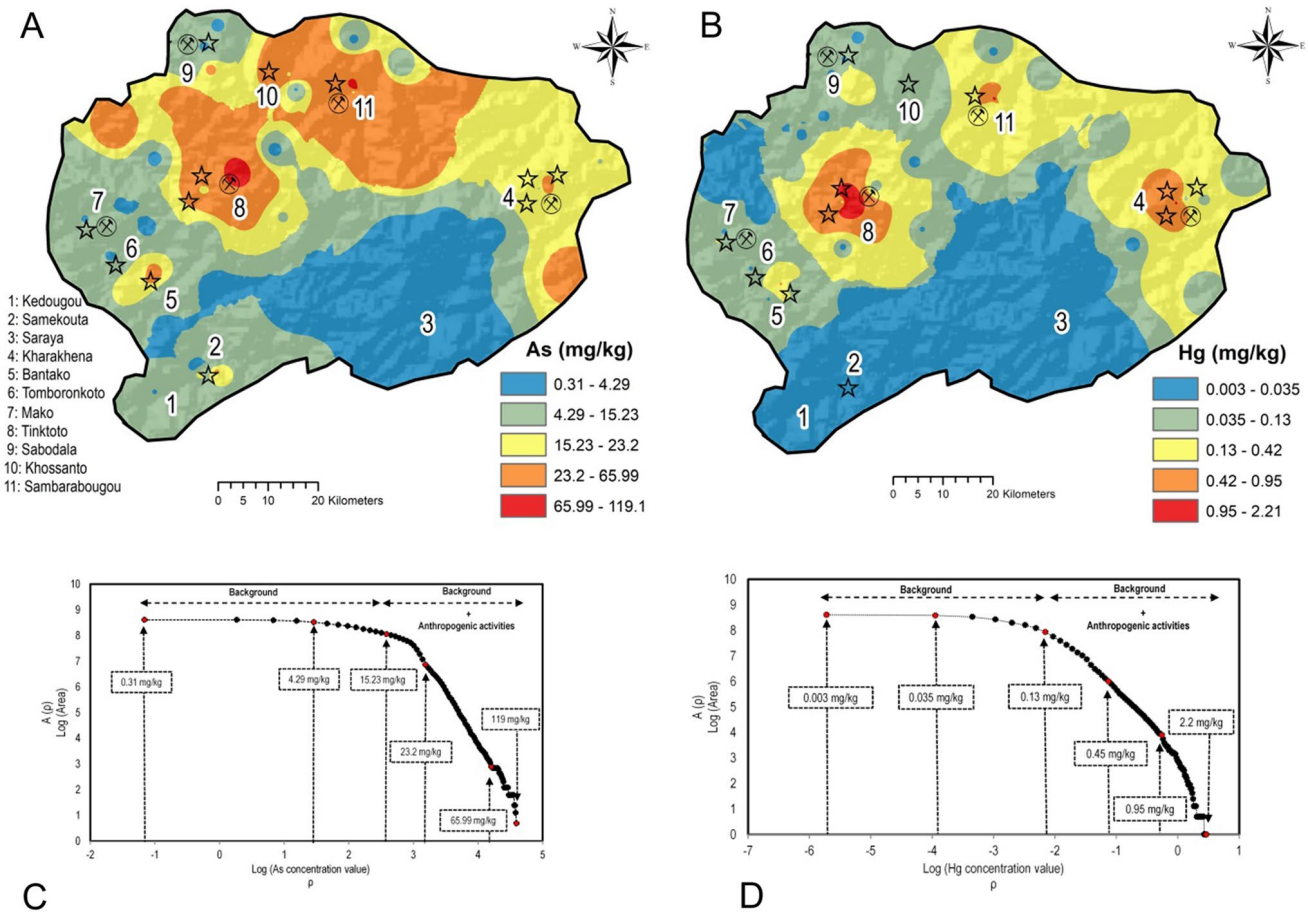
Interpolated maps of As **A** and Hg **B** elemental distribution in the survey area; ranges of concentration are based on the **C**-**A** fractal plot held bellow (**C** and **D**)

Figure 4A shows As concentration ranging from 0.31 to 119.3 mg kg^−1^, with the greatest concentration (ranging from 65.99 to 119.3 mg kg^−1^) located in the proximity of the sampling sites in the Massawa district, where industrial Au mining companies settle. The high concentration of As in topsoil may be associated with Au-bearing ore which is typically concomitant with sulphides of various elements (including As) that may be mobilised during the prospection phases and accumulate in topsoil. Treloar et al. (2015) highlighted that the Massawa Au deposit presents a first sulphide–Au mineralization associated with disseminated arsenopyrite–pyrite.

The concentration of Hg, ranging from 0.003 to 2.40 mg kg^−1^ with a mean value of 0.14 mg kg^−1^ was separated into five ranges according to C-A fractal plot (Fig. 4B, plot below). The lowest concentrations (ranging from 0.003 to 0.035 mg kg^−1^) were located in the southern part of the study area. In contrast, the greatest concentration values ranging from 0.95 to 2.21 mg kg^−1^ were roughly corresponding to the Tinkoto, Kharakhena, and Sambarabougou villages. These locations are well known because they are linked to a large number of ASGM activities where Hg is used for amalgamation in Au recovery (Niane et al., 2014). A potential explanation could therefore be that the anomalous concentrations of Hg in our study area may be related to anthropogenic input related to ASGM activities, similarly to what evidenced by other studies (e.g., Appleton et al., 2001; Sako & Nimi, 2018). The values identified in this study are comparable to those of similar surveys conducted world-wide to assess the levels of Hg in soils nearby ASGM and estimate its impact on environment and human health (Table 2).

**Table 2:** Mercury concentration (mg kg^−1^) level in different soils nearby ASGM activities

Location	Characteristics of soils	Ranges (mg kg^−1^)	Authors
*Africa*			
Senegal	Soils surrounding ASGM, Kedougou	0.003–2.21	This study
Ghana	Soils in Households nearby ASGM, Kejetia	0.096–40.97	Rajaee et al., 2015
Tanzania	Urban area near ASGM	0.05–9.2	Taylor et al., 2005
*Asia*			
China	Soil samples collected in Tongguan Au mining area	0.3–76	Feng et al., 2006
Indonesia	Soils in surrounding Artisanal Buladu Gold Mine	0.48–4.24	Mallongi et al., 2014
Thailand	In soils nearby small-scale gold mining, Phichit Province	0.038–10. 56	Pataranawat et al., 2007
*America*			
Venezuela	Soils from the Cuyuni river basin, near ASGM	0.02–0.40	Santos-Francés et al., 2011
Chile	Soils within Coquimbo region	2.4–47	Higueras et al., 2004
Bolivia	Soils in agriculture land around ASGM	0.5–48.6	Terán-Mita et al., 2013

### Multivariate analysis and elemental sources

#### Factor analysis

Robust factor analysis was computed on the 28 selected variables to highlight the geochemical characteristics that can help identify their environmental behaviour and distinguish their geogenic and/or anthropogenic origin in the survey area.

The total variance was 67.1% in a four-factor model based on the breakpoint on the scree-plot of all factors. The four factors (*F*1, *F*2, *F*3 and *F*4), accounted for 22.4, 16.2, 14.2 and 14.1% variability, respectively (Table 3).

**Table 3:** Varimax-rotated factor (three-factor model) of isometric logratio clr back-transformed variables for topsoil samples from the survey area; bold entries: loading values over |0.50|

Variables	Factors				Communalities
	F1	F1	F1	F1	
Ag	0.03	**0.82**	− 0.08	− 0.05	0.69
Al	0.03	− **0.65**	0.06	0.46	0.63
As	− 0.04	0.22	**0.83**	0.22	0.79
Au	0.2	**0.76**	0.12	0.23	0.69
Be	− 0.23	**− 0.6**	0.16	0.25	0.5
Ca	**0.75**	0.26	0.02	− 0.41	0.79
Cd	0.1	**0.53**	− 0.15	− 0.43	0.5
Co	**0.79**	0.1	− 0.16	0.39	0.82
Cr	0.17	− 0.2	0.33	**0.58**	0.51
Cu	**0.5**	0.04	0.11	**0.68**	0.72
Fe	0.06	− 0.15	**0.66**	**0.66**	0.9
Hg	0.08	**0.72**	0.3	− 0.08	0.62
K	0.34	− 0.31	− 0.25	**− 0.73**	0.81
Mg	**0.81**	− 0.17	− 0.11	− 0.34	0.82
Mn	**0.7**	− 0.06	− 0.27	0.14	0.59
Mo	− 0.17	− 0.1	0.7	0.13	0.54
Na	0.34	0.42	− 0.12	**− 0.55**	0.61
Ni	**0.79**	0.09	− 0.03	0.46	0.84
P	0.05	− 0.13	**0.78**	− 0.06	0.63
Pb	− **0.64**	− 0.1	0.19	0.23	0.51
Sb	0.1	0.04	**0.81**	0.26	0.73
Se	0.33	**− 0.52**	0.21	− 0.3	0.51
Sn	− **0.8**	− 0.09	− 0.31	− 0.06	0.74
Th	− **0.83**	− 0.36	− 0.25	0.05	0.88
Ti	− **0.51**	**− 0.5**	− 0.24	0.38	0.62
U	− **0.77**	− 0.22	− 0.14	− 0.03	0.67
V	0	− 0.24	**0.59**	**0.67**	0.85
Zn	0.36	**0.68**	0.06	− 0.17	0.62
Eigenvalues	7.3	5.79	3.66	2.04	
Total variance in %	22.43	13.18	14.17	14.11	
Cum. of total variance (%)	22.43	38.83	53.01	67.12	

In general, variables with loadings greater than the absolute value of 0.5 are assumed describing well the main composition of each factor. The four factors identified had all variables holding communalities above 0.5 (50% of variability) which highlights the strength of the models in characterising the elements interrelationships and their most likely sources (geogenic and/or anthropogenic-Table 4). The 28 elements within models were organised by positive and negative loadings and sorted in descending order (see also Table 4):

**Table 4:** Explanation of the four-factor model extracted which explains 67.12% of total variance and presumes sources patterns of elements in the study area

Factors	% of variance explained	Association of variables	Interpretation	
*F*1	22.43	Mg, Ni, Co, Ca, Mn,—(Th, Sn, U, Pb, Ti)	Th, Sn, U, Pb and Ti are likely related to Geogenic sources, likely related to Paleoproterozoic granitoids massifs of the Saraya Batholith	Figure 5A
			Mg, Ni, Co, Ca and Mn corresponds to sedimentary deposits of the Dialé-Daléma series	Figure 5A
*F*2	16.18	Ag, Au, Hg, Zn, Cd,—(Al, Be, Se, Ti)	Ag, Au, Hg, Zn and Cd related to anthropogenic contribution, likely related to artisanal gold mining activities	Figure 5B
			Al, Be, Se andTi may be correlated to granitoids massifs of the Saraya Batholith	Figure 5B
*F*3	14.17	As, Sb, P, Mo, Fe, V	As, Sb, P, Mo, Fe and V = geogenic sources, from Mako volcanic belt rich in	Figure 6A
*F*4	14.11	Cu, V, Fe, Cr,—(K, Na)	Cu, V, Fe and Cr likey featured with igneous rocks based tholeiitic formations	Figure 6B
			K and Na related to Saraya Batholith with high-K calc-alkaline series	Figure 6B

*F*_1_: Magnesium—Mg, Ni, Co, Calcium—Ca, Manganese—Mn,—(Thorium—Th, Sn, Uranium—U, Pb, Titanium—Ti).

*F*_2_: Silver—Ag, Au, Hg, Zn, Cd,—(Aluminium—Al, Be, Se, Ti).

*F*_3_: As, Sb, Phosphorus—P, Molybdenum—Mo, Iron—Fe, V

*F*_4_: Cu, V, Fe, Cr,—(Potassium—K, Sodium—Na).

The factor score distribution maps were prepared using the concentration–area (C-A) fractal function available in GeoDAS (Cheng et al, 1994; Thiombane et al., 2018).

**Fig. 5:**
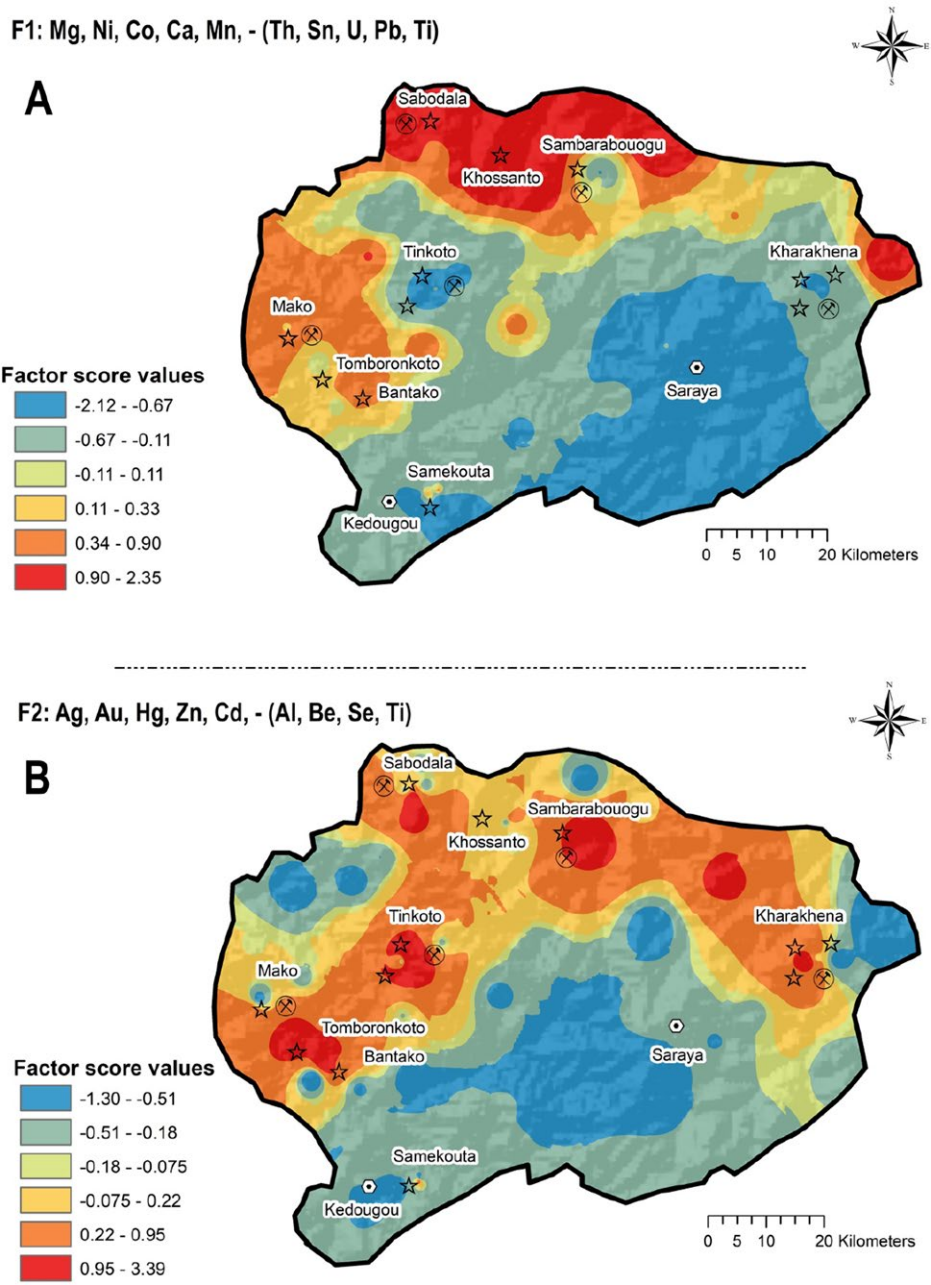
Interpolated factor score maps of the Factor 1 **A** and Factor 2 **B**

The factor scores interpolated map F1 (Fig. 5A) highlights scores ranging from − 2.12 to 2.35. Low factor score (ranging from − 2.12 to  − 0.67) are in correspondence to the Saraya area and surroundings. These low factor score reveal a dominance of Th, Sn, U, Pb, and Ti in the soils of this portion of the study area. This may be related to the underground geological features directly related to the soils above. In fact, Fall et al. (2018) described Th-U anomalies in the Paleoproterozoic granitoids massifs of the Saraya Batholith. High factor scores (ranging from 0.90 to 2.35) are located in the Northern part of the study area where the association of Mg, Ni, Co, Ca, and Mn may be related to the sedimentary deposits of the Dialé-Daléma series. Several authors (e.g., Bessoles, 1977; Hirdes & Davis, 2002; Gueye et al., 2008; Dabo & Aïfa, 2010; Lambert-Smith et al., 2016) reported that the sedimentary deposits of the Dialé-Daléma series consist of siliciclastic and minor carbonate rocks.

Figure 5B shows the factor score of variables in the F2 factor, with values ranging from − 1.30 to 3.39. High factor scores of the F2 range from 0.95 to 3.93 and are related to the positive correlation between Ag, Au, Hg, Zn, and Cu roughly located in correspondence of the villages of Tomboronkoto, Bantako, Tinkoto, Sambarabougou, Kharakhena and Samekouta. The underlying bedrock of these areas is mostly constituted of volcanic rocks with quartz vein-hosted Au. It is likely that, based on the presence of Au-mineralisation, the use of Hg for Au amalgamation in ASGM activities may be practiced in these areas (Niane et al., 2014, 2015). Indeed, the composition of the F2 factor and its geographical distribution seems to indicate an anthropogenic contribution of Hg related to activities such as ASGM practices.

**Fig. 6:**
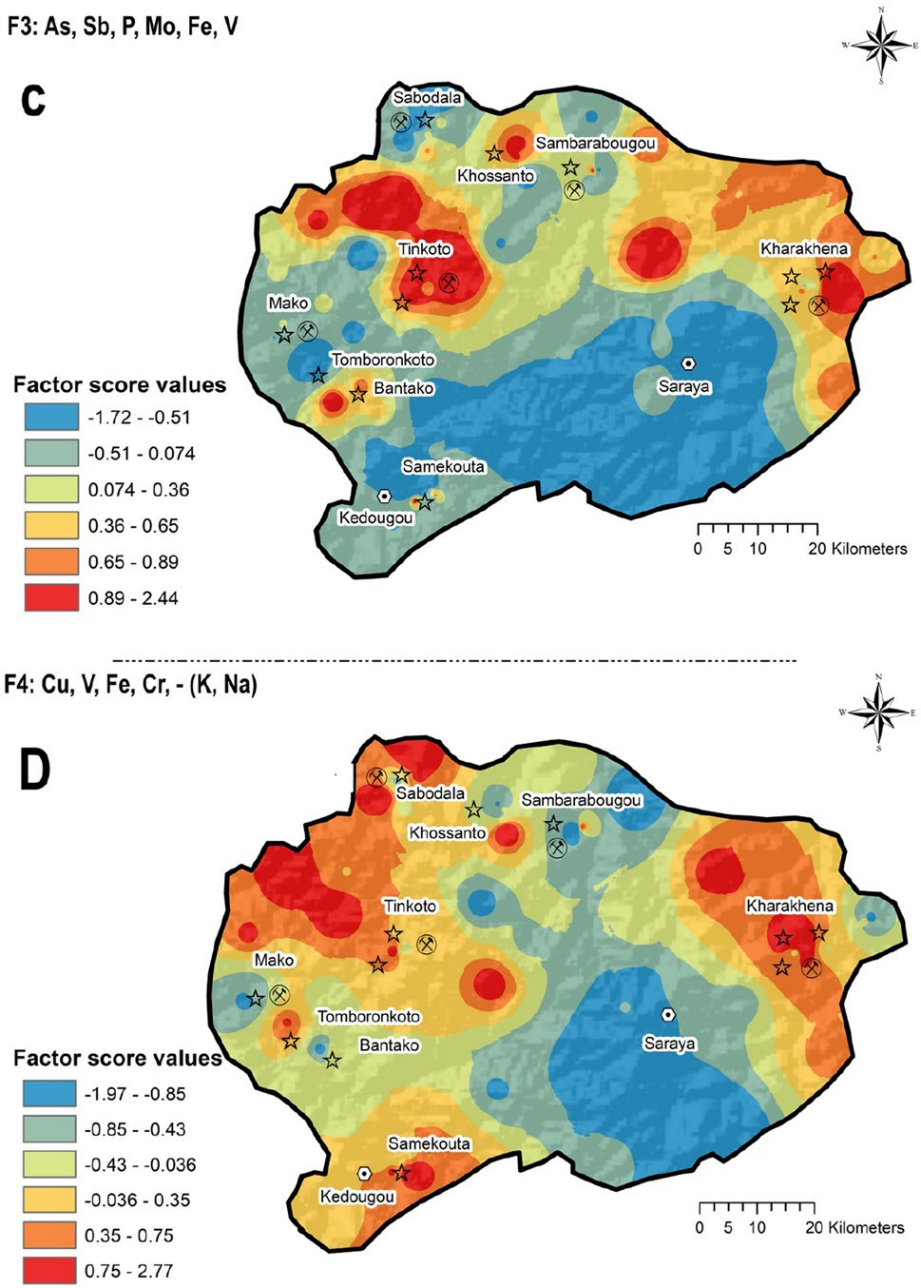
Interpolated factor score maps of the Factor 3 **C** and Factor 4 **D**

The interpolated map for the factor score 3 (Fig. 6A) shows values ranging from -1.72 to 2.44, corresponding to As, Sb, P, Mo, Fe, and V association with elevated values (ranging from 0.89 to 2.44) observed in Tinkoto and Massawa districts. This elemental association is likely to be related to the underlying geology dominated by volcanic rocks (Mako Volcanic belt), which formed most of the soils of this region. Topsoils of the area of Massawa are reported to be part of the Mako Volcanic belt, which is part of the exploration domain of LSGM, where the “Massawa Au deposits” were discovered. Treloar et al. (2015) highlighted that this section of the Mako Volcanic Belt is composed of two major styles/stages of mineralization: 1) sulphide–Au mineralization associated with disseminated arsenopyrite–pyrite and 2) quartz–stibnite + tetrahedrite veins characterised by coarse visible Au. The two stages of Au mineralization are separated by a phase of quartz–molybdenite veining. Higher factor loading of As in this association emphasizes its anomalous nature in this part of our survey area, possibly related to potential mobilization of elements by weathering and/or soil formation. Further studies would be needed to identify the potential sources of As, characterised by very high concentrations (up to 65 mg kg^−1^) in this area. Figure 6B highlights a positive Cu, V, Fe, and Cr and negative K and Na correlations which correspond to elevated (ranging from 0.75 to 2.77) and low (ranging from -1.97 to -0.85) factor score values, respectively. High factor score values can be observed on the north-western and eastern part where the Mako Volcanic Belt and Falémé volcanic deposits occur, respectively. These igneous rocks are tholeiitic formations characterised by sulphide mineralisation such as chalcopyrite (Bassot, 1987; Sylla & Ngom, 1997). Furthermore, elevated concentration of Fe (ranging 152,000 to 265,200 mg kg^−1^) were observed in topsoil samples of the eastern part of the survey area, possibly being linked to the Precambrian magnetite skarn deposits of the so-called Falémé iron district (Bassot, 1997; Schwartz & Melcher, 2004; Wade, 1985). Low factor score values instead corresponded with the Saraya district and surroundings. Fall et al. (2018) showed through regional radiometric measurements that the Saraya Batholith displayed high-K calc-alkaline series, and Bassot (1966) and Ndiaye et al. (1997) also revealed the presence of syenogranite with biotite and muscovite. The evidence seems to be consistent with K-enrichment in topsoils of this area caused by alteration and weathering phenomena of major K-bearing phases in granite, including potassic feldspars.

#### Regression analysis and contamination sources discrimination

**Fig. 7:**
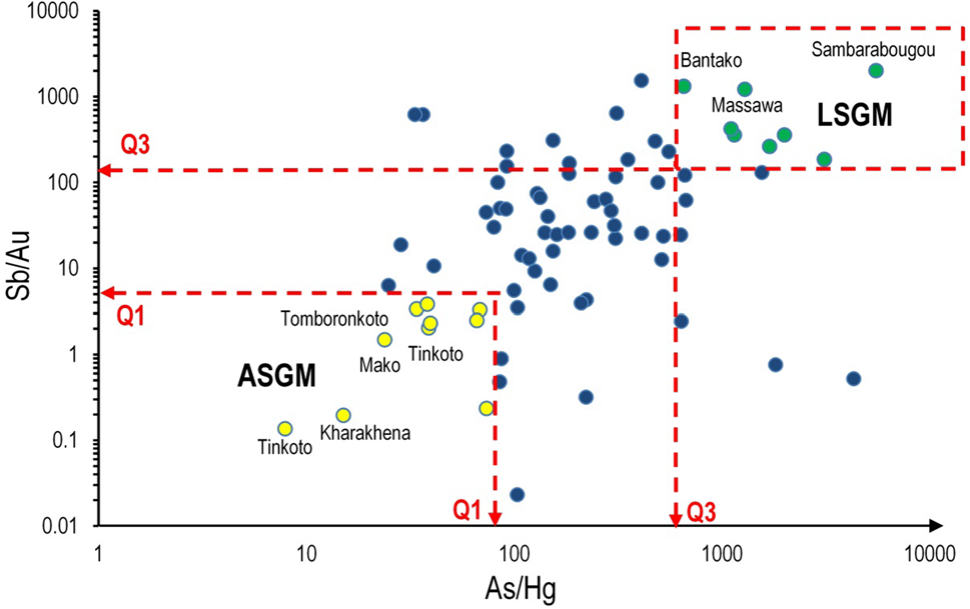
Multi-element regression plot of Sb/Au and As/Hg ratios. Q1 and Q3 symbolise first (25%) quartile and third (75%) quartile, respectively

A multi-element regression plot based on the Sb/Au and As/Hg ratios was prepared and divided in two areas (Fig. 7), with each one corresponding to a specific element dominance.

Area Q1: This area is characterised by lower values (i.e. below the Q1 values) of Sb/Au and As/Hg ratios which correspond to a dominance of Hg and Au in topsoils. Significantly, it can be observed how area Q1 values correspond mostly to sampling points taken from the areas where ASGM activities can be found. Furthermore, Pearson correlation (r) between Hg and Au was calculated for topsoils (*r* = 0.74) and bottom soils (*r* = 0.21), showing a higher correlation in the former, which suggests that Hg behaviour may be linked more to an anthropogenic input than a geogenic one. Artisanal gold mine activities could produce accumulation and redistribution in the more superficial soil horizons, providing a very likely source for Hg topsoil contamination in the surveyed area.

Area Q3: Values with greater ratios, i.e. where the ratios Sb/Au and As/Hg were above their respective third quartile (Q3), were found mostly in correspondence with the Massawa and Sambarabougou sampling points, displaying high concentrations of As and Sb. Furthermore, Pearson correlation between As and Sb in topsoil and bottom soil were calculated at (*r* = 0.52) and (*r* = 0.59), respectively. These intermediate values could be related to either geogenic and/or anthropogenic sources. It can be argued that the high concentration of As and Sb in the topsoils of Massawa and Sambarabougou may be related to both local activities (Au mining) but also weathering phenomena that could induce the enrichment/contamination of As and Sb in topsoils. Whether these natural re-mobilizations are more prominent compared to anthropogenic accumulation is difficult to deduce, though it is well known that in the Massawa and Sambarabougou areas, LGSM companies are working on Au deposits.

### PHE contamination assessment

Assessment of soil contamination/pollution on PHEs content in soils was expressed as EF and IGeo following the values and ranges available in the literature (Table 5). These values were compared to those of the Robust Compositional Contamination Index.

**Table 5:** Indices (Enrichment factor geoaccumulation index) of pollution used in this study

Indices	Ranges	Pollution levels
	< 2	Minimal
Enrichment factor (EF)	5-Feb	Moderate
Sutherland (2000)	20-May	Significant
	20–40	Very high
	> 40	Extremely high
	─	─
	≤ 0	Unpolluted
	0–1	Unpolluted to moderately polluted
	2-Jan	Moderately polluted
Geoaccumulation Index (Igeo)	3-Feb	Moderately to highly polluted
Müller (1969)	4-Mar	Highly polluted
	5-Apr	Highly to extremely polluted
	≥ 5	Extremely highly polluted

#### Enrichment factor and contamination levels

Table 6 shows the variation of background values of the considered 15 PHEs in soils of our study area, where GeoM were considered to compute the EFs.

**Table 6:** Descriptive statistic of 15 considered PHEs in bottom soils of this surveyed area; the background concentration of the 15 PHEs correspond to their respective Geometric mean (GeoM) in 18 bottom soils samples collected in areas characterised by an absent of industrial activities

Elements (mg/kg)	Cu	Pb	Zn	Ni	Co	As	Cd	Sb	V	Cr	Tl	Hg	Se	Sn	Be
Num. Samples	18	18	18	18	18	18	18	18	18	18	18	18	18	18	18
Minimum	24.02	2.61	6.7	4.7	1.4	2.2	0.0065	0.1	6.01	11.1	0.03	0.003	0.065	0.065	0.2
Maximum	99.16	21.5	38.7	95.7	57.6	115.6	0.02	1.05	387	811.8	0.08	0.146	0.3	4.3	1.4
Mean	46.48	10.26	19.3	40.4	19.3	28.44	0.0089	0.58	163.86	189.81	0.05	0.04	0.1	1.18	0.67
GeoM	42.32	8.26	15.52	25.13	10.54	14.45	0.0081	\0.44	96.53	81.87	0.04	0.02	0.09	0.64	0.56
Median	38.07	10.24	14.1	21.3	11.1	16.4	0.0065	0.53	159	122.5	0.04	0.022	0.065	0.7	0.6

**Fig. 8:**
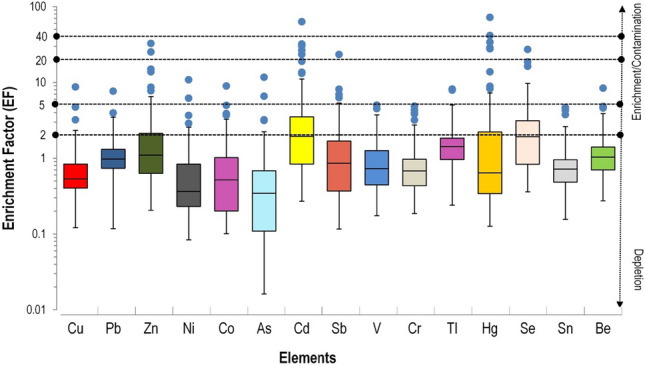
Tukey's box-and-whiskers plots representing the enrichment factor variation of 15 considered PHEs; blues dots symbolise outlier’s observations

Figure 8 shows Tukey's box-and-whisker plots of the 15 considered PHEs, organized according to their distribution towards the EF values given by Sutherland (2000).

According to its original classification in 5 ranges, it was found that V, Cr, and Sn presented the lowest EFs variation, ranging from minimal enrichment (EFs < 2) to moderate enrichment (EFs ranging from 2 to 5), with all of them presenting EFs medians and third quartile (Q3) values below 2. Vanadium, Cr, and Sn elemental distributions therefore indicate minor enrichment in topsoil, which could be confidently associated to geogenic origin.

Copper, Pb, Ni, Co, As, TI, and Be displayed EFs values ranged from minimal (EFs < 2) to significant (EFs ranging from 5 to 20) enrichment. All these elements presented medians and Q3 values below 2, indicating a moderate enrichment/contamination level, with some isolated significant enrichment (EFs range from 5 to 20) in correspondence to soils belonging to the Mako Volcanic Belt.

Enrichment factor values of Zn, Sb, and Se were found to range from minimal (EFs < 2) to very high (EF ranging from 20 to 40) enrichment/contamination level, and Zn and Se presented Q3 values above 2. The high enrichment level in topsoils could be related to both mining activities and/or weathering processes of the parental rocks which subsequently formed soils. It is well documented that Zn, Sb and Se are mainly related to volcanic rocks and they tend to accumulate on the surface (Alloway, 2013; Kabata-Pendias, 2011).

Cadmium and Hg displayed EFs factor ranging from minor (EFs < 2) to extremely high enrichment factor. Both elements show Q3 above 2 and many observations with EFs higher than 20. Furthermore, high Hg and Cd EFs were found in areas where ASGM activities occur. These observations strongly point towards both anthropogenic activities and geogenic processes: Hg-Au-amalgamation is likely to be a major Hg contamination source in ASGM (Alloway, 2013) whilst Cd-enrichment in topsoils may be related to leaching processes and sluicing of the Cd-rich in sedimentary rocks (Alloway, 2013; Kabata-Pendias, 2011).

#### Index of geoaccumulation and pollution insight

**Fig. 9:**
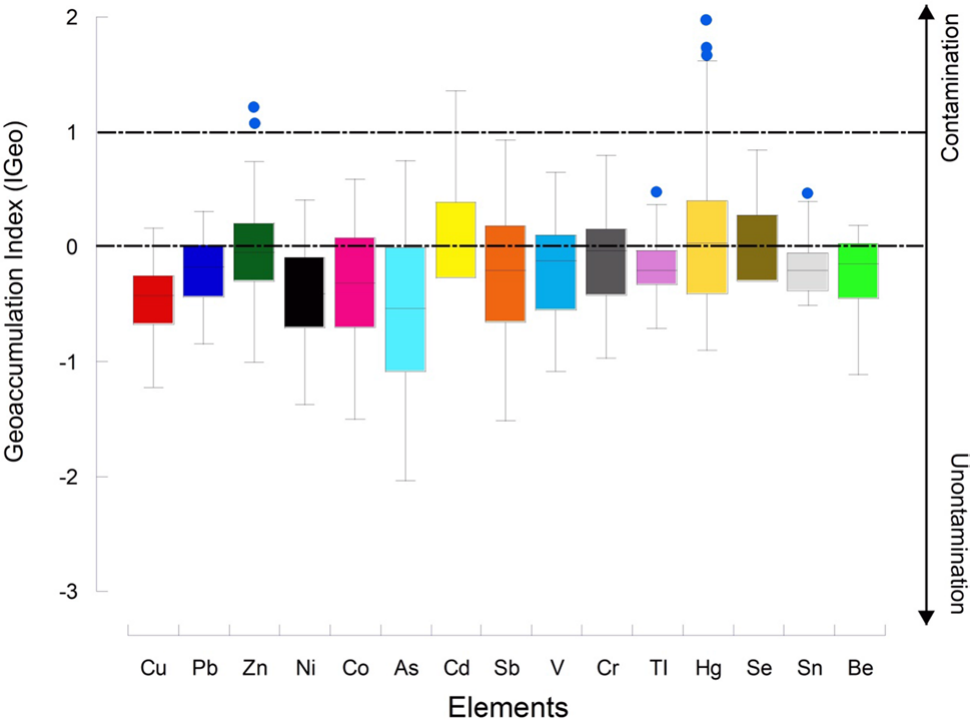
Tukey's box-and-whiskers plots representing the Geoaccumulation indices variation of 15 considered PHEs

Figure 9 shows IGeo indices of the 15 considered elements, with ranges (dashed lines) based on Muller (1969) intervals.

Apart from Zn, Cd and Hg, all other elements presented IGeo values ranging from unpolluted (IGeo < 0) to unpolluted-moderately polluted (IGeo ranging from 0 to 1). Among these elements, Pb (*Q*3 = 0.02), Co (Q3 = 0.11), As (*Q*3 = 0.03), Sb (*Q*3 = 0.23), V (*Q*3 = 0.16), Cr (*Q*3 = 0.19), Se (*Q*3 = 0.32), and Be (*Q*3 = 0.03) showed Q3 values ranging from 0 to 1. Based on the IGeo, it can be concluded that Cu, Pb, Ni, Co, As, Sb, V, Cr, TI, Se, Sn, Sn, and Be are characterised by a low pollution level in the study area. In contrast, Zn, Cd, and Hg presented IGeo values ranging from unpolluted (IGeo < 0) to moderately contaminated (IGeo ranging from 1 to 2). In particular, Hg presented the highest IGeo values in correspondence to sampling sites near ASGM activities. Once more, moderate Hg pollution level in the surveyed soils seems to be related to ASGM activities, where Au-amalgamation with Hg is carried out.

#### Robust compositional contamination index (RCCI)

In order to highlight the relative compositional contamination level, RCCI was computed based on 15 considered PHEs. The results were mapped to assess the relative spatial abundance of said elements to distinguish a more realistic contamination loading in the study area.

**Fig. 10:**
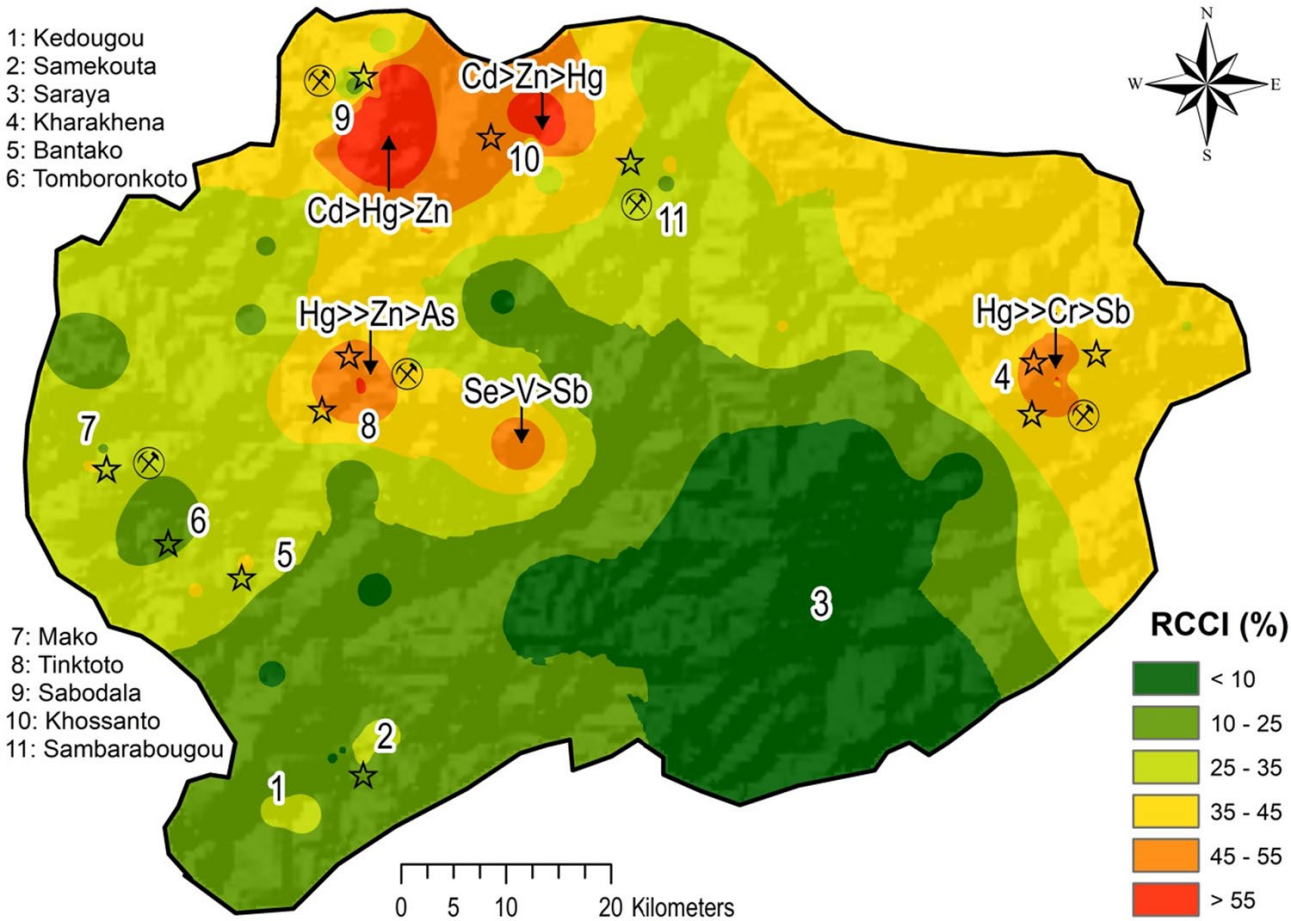
RCCI Interpolated map of the 15 considered PHEs. Red colour symbolises highest relative contamination level

The RCCI map (Fig. 10) showed low index (values < 25%) in the southern part of the study area where the two main cities (Kedougou and Saraya) are located. This region seems to be relatively uncontaminated with regards to the considered elements.

Medium RCCI values (ranging from 25 to 45%) were mapped roughly in the Western (Tomboronkoto (6), Bantako (5) and Mako (7)) and central parts (Sambarabougou (11)) part of the study area. In fact, it is indicative that ASGM activities can be found in these areas, potentially giving rise to relatively significant release of PHEs in the environment.

The high RCCI values (ranging from 45 to 55%) were found to correspond to the Kharakhena (4) and Massawa (8) districts. In particular, the high RCCI in Kharakhena was also accompanied by high GeoM of Hg, Cr, and Sb. Unsurprisingly, this area is characterised by a considerable number of ASGM activities that are likely to use Au-Hg amalgamation techniques for their mining activities. On the other hand, the high RCCI in the Massawa district (dominance of Se, V, and Sb) could be related to a mix of geogenic/anthropogenic sources, potentially related to industrial and mining activities located in this area.

The highest spatial index (RCCI values up to 55%) were found in the northern part of the study area, specifically in Sabodala (9) (Cd > Hg > Zn), Khossanto (10) (Cd > Zn > Hg) and Tinkoto (8) (Hg >  > Zn > As) districts. In Sabodala and Khossanto, the GeoM of Cd, Hg and Zn prevailed in the RCCI computation and these areas are also characterised by the presence of large ASGM activities. The levels of contamination in these two areas are very likely to be related to anthropogenic activities such as artisanal Au mining. Moreover, Sabodala hosts the largest LSGM Company of the Kedougou region, whose activities might explain the high levels of Cd and Zn in soils of this area. In contrast, Tinkoto hosts the largest ASGM activities of the Kedougou region (ANDS, 2015; Niane et al., 2015); here results were characterised by high GeoM of Hg, followed by Zn and As, which may be related to use of Hg in artisanal Au mining activities, but also potentially related to mineral impurities, as As and Zn are usually found as major impurity elements in native Au mineralization (Treloar et al., 2015).

## Conclusion

This study focused on providing a better understanding of multi-element geochemistry and their source patterns by means of geostatistical computations and spatial mapping, in order to identify potential contamination and enrichment levels of the 15 PHEs in soils of the Kedougou region.

The multivariate and integrated approach revealed the correlation between variables and facilitated the identification of the main sources of elements in the surveyed region. Robust factor analysis based on transformed data determined the elemental relationships with the main geological features but also the potential contribution of anthropogenic activities in the Kedougou region. In particular, the sedimentary deposits of the Dialé-Daléma series were associated to the elemental association of Mg, Ni, Co, Ca, and Mn, whilst K, Na, Th, Sn, U, Pb, and Ti elemental association were linked with granitoids massifs of the Saraya Batholith deposits. Mako and Falémé volcanic rocks were identified as the geogenic sources for Ag, Au, Zn, Cd, Sb, P, Mo, Fe, Cu, V, Fe, Cr. More importantly, regression analysis and elemental spatial distribution identified As and Hg anomalies associated with anthropogenic activities such as LSGM and ASGM in Kedougou.

The topsoil contamination and/or enrichment levels of 15 considered PHEs ranged widely. Enrichment Factor (EFs) and IGeo computations confirmed that Hg contamination is high in the surveyed area, and occurs mostly where ASGM activities are located, pointing strongly to this activity as a source, likely as a result of the Hg-Au-amalgamation techniques used in such ASGM activities. Further emphasis on this type of contamination was provided using RCCI, which was mapped to reveal spatial variability. High RCCI values were found in correspondence with the Sabodala, Khossanto and Tinkoto areas, once again where LSGM and ASGM activities are located.

The findings from this investigation strongly advocate follow up studies in clearly identified and discrete areas displaying high concentration of PHEs and contamination levels: this would allow a more detailed and thorough assessment of PHEs, and their accumulation in different environmental matrices such as sediments as well as biological samples (e.g. fishes, cultivated crops, and human hair). A comprehensive evaluation of human health risk due to direct and indirect exposure is strongly recommended, particularly when considering that PHEs such as Hg and As are able to bioaccumulate and/or biomagnify in food chains upon which humans are dependent.
